# Metabolic and functional characterization of effects of developmental temperature in *Drosophila melanogaster*

**DOI:** 10.1152/ajpregu.00268.2016

**Published:** 2016-12-07

**Authors:** Mads F. Schou, Torsten N. Kristensen, Anders Pedersen, B. Göran Karlsson, Volker Loeschcke, Anders Malmendal

**Affiliations:** ^1^Department of Bioscience, Aarhus University, Aarhus C, Denmark;; ^2^Department of Chemistry and Bioscience, Aalborg University, Aalborg East, Denmark;; ^3^The Swedish NMR-Centre, University of Gothenburg, Gothenburg, Sweden; and; ^4^Department of Cellular and Molecular Medicine, University of Copenhagen, Copenhagen N, Denmark

**Keywords:** nuclear magnetic resonance metabolomics, developmental acclimation, environmental stress, extreme temperatures, plastic responses, sexual dimorphism, thermal performance curve, thermal resistance

## Abstract

The ability of ectotherms to respond to changes in their thermal environment through plastic mechanisms is central to their adaptive capability. However, we still lack knowledge on the physiological and functional responses by which ectotherms acclimate to temperatures during development, and in particular, how physiological stress at extreme temperatures may counteract beneficial acclimation responses at benign temperatures. We exposed *Drosophila melanogaster* to 10 developmental temperatures covering their entire permissible temperature range. We obtained metabolic profiles and reaction norms for several functional traits: egg-to-adult viability, developmental time, and heat and cold tolerance. Females were more heat tolerant than males, whereas no sexual dimorphism was found in cold tolerance. A group of metabolites, mainly free amino acids, had linear reaction norms. Several energy-carrying molecules, as well as some sugars, showed distinct inverted U-shaped norms of reaction across the thermal range, resulting in a positive correlation between metabolite intensities and egg-to-adult viability. At extreme temperatures, low levels of these metabolites were interpreted as a response characteristic of costs of homeostatic perturbations. Our results provide novel insights into a range of metabolites reported to be central for the acclimation response and suggest several new candidate metabolites. Low and high temperatures result in different adaptive physiological responses, but they also have commonalities likely to be a result of the failure to compensate for the physiological stress. We suggest that the regulation of metabolites that are tightly connected to the performance curve is important for the ability of ectotherms to cope with variation in temperature.

the thermal environment is central for the abundance and distribution of ectotherms and much attention has been directed toward the potential of ectotherms to adapt to current and future thermal conditions (2, 15, 16, 20, 22, 42). Predicting the future distributions of ectotherms in a warmer, but also more variable, environment with more extreme thermal episodes at both ends of the thermal scale can be addressed by studying the ability to adapt through evolutionary, behavioral, or plastic responses (23). A number of recent studies suggest that some species of frogs (17), phytoplankton (24), copepods (27, 28), and insects (22) are evolutionarily constrained when it comes to coping with extreme environments due to, e.g., lack of adaptive genetic variation. Thus current and future species distributions will partly depend on migration, behavioral thermoregulation, and physiological plastic responses; the latter being the focus of this study.

Acclimation is a form of physiological plastic response that organisms induce in response to changes in the environment. Laboratory studies on ectotherms investigating acclimation and the physiological changes induced during acclimation typically do so by exposing test organisms to short-term cold or hot temperatures and subsequently studying the consequences on thermal resistance and molecular phenotypes (11, 26, 39, 41, 44, 62, 64, 65, but see 7, 36). Such studies have provided novel insights into the molecular aspects of the acclimation response and have contributed to our knowledge on associations between the genotype and the phenotype. However, in their natural habitats organisms are exposed to thermal variation throughout the entire life cycle and to thermal stress for extended periods, e.g., during cold winters or hot summers (25, 50, 57), whereby molecular characteristics of long-term exposure to different thermal environments is of ecological importance (30). This includes extreme environments that may induce more physiological stress than beneficial acclimation. A linear increase in ambient temperature may result in nonlinear reaction norms at molecular levels; for example, a small increase in temperature at 20°C may not be perceived in the same way as a small increase in temperature at 30°C. Molecular reaction norms across the thermal range can be investigated by quantifying a molecular phenotype such as gene expression at each temperature. This enables grouping of genes into different categories of reaction norms and quantifying levels of molecular plasticity, furthering our understanding of the underlying components of long-term thermal acclimation (7).

Metabolomics has been implemented to study consequences of age, sex, and genotype in *D. melanogaster* and encompasses the power to detect biologically relevant changes in the metabolome (21). Metabolomics may thus prove to be an important tool when filling the gap between the transcriptome and the functional phenotype (47). In organisms such as springtails, fruit flies, echinoderms, and fish, metabolomics has provided insight into hot and cold adult acclimation responses (39, 43, 59, 60, 70, 71). An increase in sugars, polyols, and free amino acids has been related to long-term cold acclimation in both adult and larva *D. melanogaster*, as well as in winter-acclimating individuals of the codling moth *Cydia pomonella* (10, 32, 52). Proline in particular has been shown to be a central compound for the freeze tolerance of the drosophilids *D. melanogaster* and *Chymomyza costata* as well as the beetle *Alphitobius diaperinus* (33, 34). In other species such as the flesh fly *Sarcophaga crassipalpis*, glycerol is an important polyol during cold acclimation (38, 40, 73), whereas this does not seem to be the case in *D. melanogaster* (29, 33). As a response to short-term cold acclimation, sugars such as trehalose and glucose have been suggested to be important (43, 64). For a comprehensive overview, and in particular more information on short-term acclimation responses, which is not the focus of this study, we refer to Purać et al. (47). In comparison, studies investigating metabolomics of heat acclimation in insects are sparse (47) and especially metabolomics studies on long-term temperature acclimation are yet to be performed (18, 19).

Here we exposed *D. melanogaster* to 10 different developmental and adult rearing temperatures spanning most of the temperature range (12 to 32°C) where this species can complete its lifecycle. We obtained detailed functional phenotypic and metabolite profiles of adult male and female flies from each thermal regime. The inclusion of extreme developmental temperatures and parallel assessments of egg-to-adult viability and developmental time as a measure of stress perceived allows us to separate the metabolites in two distinct groups. The “beneficial” changes in metabolites, which form part of the thermal acclimation response, and the “costly” metabolites, which we hypothesize change as a result of environmental stress and lead to a departure from cellular homeostasis. By doing this we gain key ecological knowledge on the association between temperature exposure and functional and molecular phenotypes. This allows us to investigate how ectotherms perceive and plastically adapt to different thermal conditions and allows us to identify the physiological causes of the strong functional benefits and costs typically associated with thermal acclimation (35). Our goal was to provide a comprehensive analysis of the physiological background for costs and benefits associated with developmental temperature on thermal resistance. We discuss our data based on three a priori hypothetical types of reaction norms for the metabolome across developmental temperatures: *1*) No change in the metabolome across temperatures, indicating that the metabolome is unrelated to the developmental temperature; *2*) linear change in the metabolome across temperatures, indicating that the metabolome is a component of the physiological thermal response of the organism; and *3*) a U-shaped (or bell-shaped) change in the metabolome across temperatures, indicating a shared effect of environmental stress at extreme low (12°C and 15.5°C) and high (31°C and 32°C) developmental temperatures.

## MATERIALS AND METHODS

### 

#### Acclimation and rearing procedure.

The laboratory population used in this study was established from the offspring of 589 *D. melanogaster* mated females (five males and five females from each) caught in Denmark in 2010 [for details see Schou et al. (56)]. At the time the experiment was initiated, the population had been reared in the laboratory for ~45 generations. Before the experiment, the population was maintained at 20°C at a 12:12 light/dark (L/D) photoperiod and reared on a standard *Drosophila* medium composed of yeast, oatmeal, sugar, and agar. Parental flies used for egg production of experimental flies were density controlled during development. When the parental flies were 4 days of age they were transferred to bottles with the standard *Drosophila* medium but with a relatively high agar concentration (3%) to ensure that eggs were deposited on the surface of the medium to ease removal of the eggs (55). The following day parental flies were removed from the medium (after 16 h of egg-laying) and eggs were washed off the surface of the medium and distributed into vials in groups of exactly 40 eggs. This methodology was used to ensure random grouping of eggs into vials (55). At least 20 vials with eggs were distributed to each of 10 constant developmental temperatures: 12, 15.5, 18, 20, 22, 25, 27, 29.5, 31, and 32°C. We expected a lower survival rate at 12, 31, and 32°C, and therefore we set up 10 additional vials at these temperatures. All acclimation regimes had a 12:12 L/D photoperiod. At the day of emergence flies were anesthetized with CO_2_, separated into sexes, mixed among vials, and relocated to their respective acclimation regime. For a subset of these vials, we counted the number of emerged adults to assess the proportion of eggs developing into adult flies (egg-to-adult viability) and developmental time from the egg to the adult life stage.

#### Thermal tolerance assays.

For each developmental temperature, male (*n* = 20) and female (*n* = 20) flies of 2-3 days of age were tested for their critical thermal minimum (CT_min_) and their critical thermal maximum (CT_max_). CT_min_ and CT_max_ are proposed ecologically relevant measures of cold and heat tolerance (1, 49, 57, 66). Flies were placed individually into sealed 6-ml glass vials and submerged into a glass tank containing a 20°C liquid. The transfer of flies to glass vials and experimental initiation took place at 20°C and lasted no more than 15 min. When the CT_max_ was assessed, the glass tank contained water, where the temperature was increased with a rate of 0.1°C/min. Conversely when the CT_min_ was assessed, the glass tank contained a mixture of ethylene glycol and water (1:1 vol/vol), and the temperature was decreased with a rate of 0.1°C/min. The flies were continuously monitored in intervals of 2–3 min, and the temperature, where no movement could be induced with a flashlight and a gentle knocking on the vials with a stick, was noted as the upper or lower thermal limit (CT_max_ and CT_min_). We interpret a high CT_max_ and a low CT_min_ as indicating high heat and cold tolerance, respectively. A subset of the phenotypic results obtained in these assays, as well as egg-to-adult viability and developmental time, has been published elsewhere, where the focus was on changes in the proteome across those three developmental temperatures (36). This subset includes male flies developed at 12, 25, and 31°C.

#### Sample preparation for NMR.

For all developmental temperatures we prepared five replicates of 40 pooled flies per sex for nuclear magnetic resonance (NMR) spectroscopy. Flies were snap frozen at 3 days of age and kept at −80°C. Samples were mechanically homogenized with a Kinematica, Pt 1200 (Buch & Holm, Herlev, Denmark) in 1 ml of ice-cold acetonitrile (50%) for 45 s. Hereafter samples were centrifuged (10,000 *g*) for 10 min at 4°C, and the supernatant (900 μl) was transferred to new tubes, snap frozen, and stored at −80°C. The supernatant was lyophilized and stored at −80°C. Immediately before NMR measurements, samples were rehydrated in 200 ml of 50 mM phosphate buffer (pH 7.4) in D_2_O, and 180 ml were transferred to a 3-mm NMR tube. The buffer contained 50 mg/l of the chemical shift reference 3-(trimethylsilyl)-propionic acid-D4, sodium salt (TSP) and 50 mg/l of sodium azide to prevent bacterial growth.

#### NMR experiments.

NMR measurements were performed at 25°C on a Bruker Avance III HD 800 spectrometer (Bruker Biospin, Rheinstetten, Germany), operating at a ^1^H frequency of 799.87 MHz, equipped with a 3-mm TCI cold probe. ^1^H NMR spectra were acquired using a single-90°-pulse experiment with a Carr-Purcell-Meiboom-Gill (CPMG) delay added to attenuate broad signals from high-molecular-weight components. The total CPMG delay was 194 ms and the spin-echo delay was 4 ms. The water signal was suppressed by excitation sculpting, potentially masking changes in metabolites (mostly sugar units) resonating in this region. A total of 128 transients of 32 K data points spanning a spectral width of 20 ppm were collected, corresponding to a total experimental time of 6.5 min.

#### Statistical analyses of phenotypic traits.

We investigated the effects of thermal regime (developmental temperature) and sex on CT_max_ and CT_min_ using linear models. The models had either CT_max_ or CT_min_ as a response variable and consisted of the predictor parameters sex (male or female) and developmental temperature (continuous) as well as their interaction. Both CT_min_ and CT_max_ models fulfilled assumptions for parametric analyses. We performed sequential model reduction and model comparisons using *F*-tests to find the minimal adequate model and to obtain *P* values for the respective predictors. Egg-to-adult viability was modeled using a logistic regression with developmental temperature as the sole predictor. Developmental temperature was modeled as a quadratic effect (continuous) as the performance curve of egg-to-adult viability across thermal regime is nonlinear (57). Several data points from the developmental temperatures 12°C and 32°C were highly influential according to Cook’s statistic, but we found this to be of biological importance for the fit and therefore maintained them in the model. We detected overdispersion in the model and corrected the standard errors using a quasigeneralized linear regression (74). The change in developmental time across developmental temperatures was modeled with a Poisson generalized linear mixed model in the R-package lme4 (3). We used a mixed model with vial as a random effect to account for possible vial effects. Developmental temperature was included as a quadratic effect (continuous) to allow for the developmental time to increase at high stressful temperatures (14), while sex (male or female) was included as a factorial effect. A group of outliers caused the assumption of normality of residuals to be violated. The outliers were spread across many temperatures and in all cases represented some highly delayed flies, relative to the mean of the given developmental temperature. As the model allowed for a good parametric fit of the observed effect of temperature, we chose to retain this model despite the deviation from normality. The minimal adequate model and *P* values for the fixed effects of egg-to-adult viability and developmental time were obtained using sequential model reduction and by model comparisons using maximum likelihood ratio tests. All statistical analyses of functional phenotypes were performed in R (48).

#### NMR data and analyses.

The spectra were processed using iNMR (http://www.inmr.net). An exponential line-broadening of 0.5 Hz was applied to the free-induction decay before Fourier transformation. All spectra were referenced to the TSP signal at −0.017 ppm and automatically phased, and baseline was corrected. The spectra were aligned using *i*coshift (54). The region around the residual water signal (4.85–4.67 ppm) was removed in order for the water signal not to interfere with the analysis. The high- and low-field ends of the spectrum, where no signals except the reference signal from TSP appear, were also removed (i.e., leaving data between 9.5 and 0.5 ppm). The spectra were normalized to total intensity to suppress separation based on variations in amount of sample. Metabolite assignments were done based on chemical shifts only, using earlier assignments and spectral databases previously described (13, 39, 45), and comparison with *Drosophila* metabolites were identified by mass spectrometry (8).

All multivariate analyses were carried out on Pareto scaled data (12) using the SIMCA13 software (Umetrics, Malmö, Sweden). Principal component analysis (PCA) was performed to assess the overall temperature and sex dependence of the metabolome. To further investigate the differentiation in the metabolome between sexes across developmental temperatures, we performed a PCA based on the difference between individual female sample spectra and the median male spectrum at each temperature as well as the difference between median female spectrum and individual male sample spectra at each temperature. In contrast to all the other analyses in this study the intensities were not centered here, and therefore the deviation from zero in the scores corresponds to the sex difference. The change in differentiation across developmental temperatures was modeled in a linear model with developmental temperature as a cubic term. The inclusion of developmental temperature as a cubic term was necessary to fulfill assumptions of parametric analyses.

Although a PCA is highly informative when analyzing the difference in metabolite profiles across the thermal acclimation gradient, additional variation can be extracted from the metabolite profiles by separating the different combinations of sex and temperature regimes using orthogonal projection to latent structures discriminant analysis (O2PLS-DA) (67). Hierarchical cluster analysis (HCA) of the Euclidean distance between the O2PLS-DA scores for each combination of developmental temperature and sex was carried out using Ward’s method in R (48) to enable visualization of the differentiation in a dendrogram. The O2PLS-DA models were validated by cross validation. Randomly chosen groups of samples were left out to predict group membership for the excluded samples until predicted values had been obtained for all samples.

To investigate the predictability of the male and female metabolomes from the two life history traits egg-to-adult viability and developmental time, as well as from the developmental temperatures, we used orthogonal projections to latent structures (OPLS) models (68, 69). We modeled the dependence of the metabolome on the developmental temperatures using two approaches based on our prior hypotheses: *1*) a linear change with developmental temperature, tested by correlating intensities with developmental temperatures (linear prior); and *2*) a U-shaped (or bell-shaped) change in intensities across developmental temperatures, tested by modeling a fit of the intensities on a categorical variable with two states, intermediate (18–29.5°C) and extreme (12, 15.5, 31, and 32°C) developmental temperatures (U-shape prior). This categorical variable is a simple representation of metabolite changes that are dependent on the deviation from intermediate nonstressful developmental temperatures rather than the absolute temperature value. The OPLS models were validated by cross validation. All samples for each combination of sex and temperature were left out one at a time, so that the predicted parameter for that combination of sex and temperature was only based on the relationship between that parameter and the metabolite concentration for the other samples.

To avoid restricting the analyses to our prior hypotheses on the relationship with developmental temperature, we also used the results from the PCA described above to infer the overall patterns of metabolite change across developmental temperatures. We first rotated the principal components to find the component that showed the most linear response to the developmental temperatures, which then allowed us to identify the shape of the component that explained as much of the remaining variation as possible. For both male and female flies, the first rotated component was rather linear (linear component), whereas the second rotated component was U-shaped (U-shape component).

Given that the rotated components from the PCA, as well as our prior hypotheses, dictate the potential for a linear or a U-shaped reaction norm of individual metabolites across developmental temperatures, we aimed at identifying which specific metabolites conformed to the two reaction norms. First, we correlated individual metabolite intensities with the linear component and U-shape component from the rotated PCA. To investigate whether the same pattern was observed when using our prior hypotheses, we correlated the individual metabolite intensities with OPLS component scores of the overall metabolome in both the linear prior model and the U-shape prior model. The same type of correlation was performed on the OPLS component scores of the overall metabolome on egg-to-adult viability and developmental time. All correlations were performed on each sex separately, except for egg-to-adult viability. Finally, we also tested which metabolites correlated with the overall differences in the metabolome between sexes. The difference between sexes was determined as the first component in a PCA of the spectral differences between male and female samples (described in *NMR data and analyses*). The significant correlations were calculated using peak correlations within the integration range used to assign the metabolite (Table 1) and verified by looking at the correlation of other characteristic chemical shifts from that metabolite (Table 1). Significant spectral correlations were identified by applying sequential Bonferroni correction (*P* < 0.05) for an assumed total number of 100 metabolites. The correlations were performed in MATLAB (The MathWorks, Natick, 2015).

**Table 1.** ^1^H NMR chemical shifts and integration range used for correlations

Metabolite	Chemical Shifts, ppm	Integration Range, ppm
Nicotinamide ribotide	9.59, 9.33, 8.99	9.595, 9.581
NAD+	9.32, 9.13, 8.82, 8.42, 6.08	9.146, 9.128
NADP+	9.28, 9.09, 8.82, 8.40, 8.13	8.832, 8.810
AMP	8.60, 8.25, 6.13	8.609, 8.584
Glucose	5.21	5.213, 5.207
Maltose	5.40, 5.22	5.406, 5.398
Mannose	5.17	5.174, 5.169
Galactoside*	4.46, 4.18, 3.93, 3.76, 3.61	4.456, 4.448
Fatty acid	5.31, 1.28, 0.89	5.325, 5.288
3-Hydroxykynurenine	7.44, 6.89, 6.69	6.704, 6.677
Acetate	1.93	1.933, 1.930
Alanine	1.47	1.474, 1.460
β-Alanine	3.16, 3.54	2.552, 2.530
Arginine	1.91, 1.73	1.742, 1.723
Asparagine	2.93, 2.83	2.930, 2.916
Aspartate	2.80, 2.65	2.796, 2.785
Glutamate	2.34, 2.12, 2.05	2.360, 2.320
Glutamine	2.44, 2.12	2.460, 2.420
Histidine	7.78, 7.05	7.055, 7.043
Hydroxyisovalerate	2.34, 1.24	1.248, 1.239
Isoleucine	1.00, 0.92	1.007, 0.989
Lactate	4.10, 1.31	1.321, 1.306
Leucine	1.73, 0.95	0.962, 0.930
Methionine sulfoxide	3.88, 3.01, 2.74, 2.32	2.739, 2.734
Phenylalanine	7.41, 7.31	7.424, 7.401
Phosphocholine	4.15, 3.58, 3.21	4.163, 4.138
Proline	2.12, 2.01	2.039, 1.995
Tryptophan	7.72, 7.52, 7.31	7.729, 7.711
Tyrosine	7.18, 6.89	7.194, 7.169
Valine	1.03, 0.98	0.987, 0.970

Identity of the metabolites was verified using all the listed chemical shifts. Signal intensities used for the correlations presented in Fig. 7 were calculated as the total intensity within the integration range. Note that some of the signals used to estimate intensities of different metabolites overlap with other signals.

## RESULTS

### 

#### Thermal resistance, egg-to-adult viability, and developmental time.

**Fig. 1.**
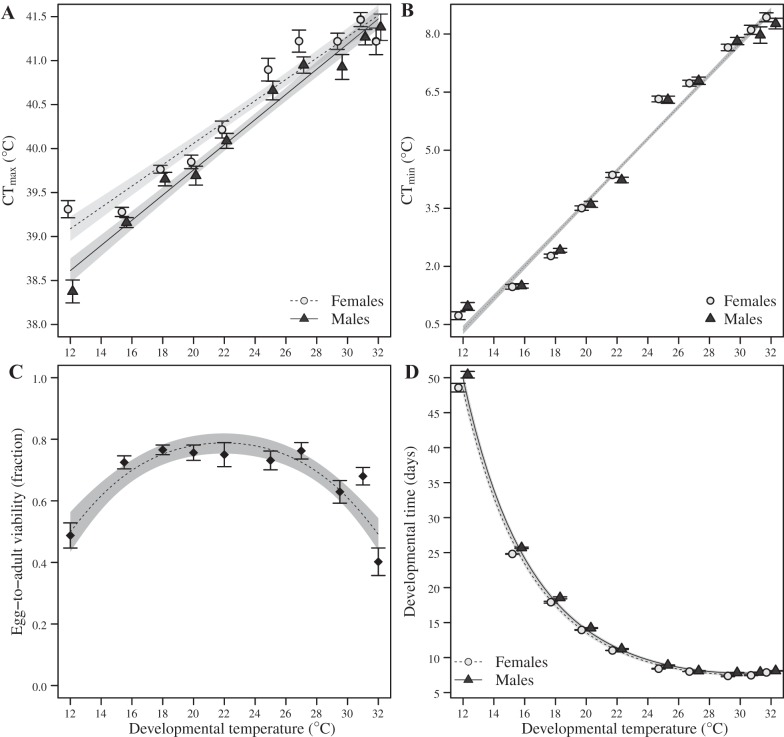
Functional phenotypes across developmental temperatures. Critical thermal maximum (CT_max_) (*A*) and critical thermal minimum (CT_min_) (*B*) as well as egg-to-adult viability (*C*) and developmental time (*D*) across thermal regimes. Flies developed at 10 different thermal regimes from egg to adult and remained there until their thermal tolerance limits were assessed at 2-3 days of age. Both CT_max_ and CT_min_ were linearly and positively correlated with developmental temperature, whereby heat tolerance was increased in flies that developed at higher temperatures, whereas cold tolerance was increased in flies developed at lower temperatures. Egg-to-adult viability had a quadratic norm of reaction as flies developed at temperatures below 15°C and above 27°C had a decreased survival. We did not register sex-specific egg-to-adult viability, and therefore use diamonds as points in the display (*C*). Developmental time decreased with increasing temperatures. Minimal adequate models from the statistical analyses are plotted with the shaded area representing the 95% confidence interval. Parametric bootstrapping was used to obtain the confidence interval of the fitted line for developmental time, which was modeled with a mixed model. Because we found no effect of sex in CT_min_, only one line representing the pooled data from both sexes has been plotted. Error bars are means ± SE.

We observed a linear relationship between developmental temperature and thermal tolerances (Fig. 1, *A* and *B*; Table 2). Cold tolerance, measured as CT_min_, was ~8°C lower in flies acclimated at 12°C compared with flies acclimated at 32°C, with no effect of sex (Fig. 1*B*; Table 2). In comparison, CT_max_ (heat tolerance) showed a very similar pattern, but with a total increase of only 2°C in flies acclimated at 32°C compared with flies acclimated at 12°C, and overall females were more heat tolerant than males (Fig. 1*A*; Table 2). The sex differentiation in CT_max_ was highest at low developmental temperatures and decreased with increasing developmental temperatures (Fig. 1*A*; Table 2). Egg-to-adult viability assessed across developmental temperatures had a quadratic shape with ~40 to 50% survival at the extremes (12 and 32°C) and with ~60 to 80% survival at the intermediate temperatures (Fig. 1*C*; Table 2). Developmental time decreased from ~49 days at 12°C to ~7.5 days at 29.5°C (Fig. 1*D*). There was no interaction between sex and temperature for developmental time, but males did develop significantly slower than females (Fig. 1*D*; Table 2).

**Table 2.** Statistics of functional phenotypes

Trait	Parameter	Estimate	SE	*F*_(d.f.)_/*χ^2^*_(d.f.)_	*P* Value
CT_min_	Intercept	−4.578	0.010		
	Developmental temperature	0.412	0.004	9852.20_(1,434)_	<0.001†
	Sex			0.19_(1,434)_	0.662
	Sex·Developmental temperature			1.73_(1,433)_	0.189
CT_max_	Intercept	37.601	0.130		
	Developmental temperature	0.123	0.005		
	Sex	−0.692	0.184		
	Sex·Developmental temperature	0.019	0.008	6.79_(1,349)_	0.009*
Egg-to-adult viability	Intercept	0.66	0.06		
	Developmental temperature	−0.81 (−4.85)	0.50 (0.53)	212.53_(2)_	<0.001†
Developmental time	Intercept	2.55	0.01		
	Developmental temperature	−26.56 (9.61)	0.28 (0.25)	501.24_(2)_	<0.001†
	Sex	0.03	0.01	9.67_(1)_	0.002*
	Sex·Developmental temperature			0.24_(2)_	0.930

Results from the statistical analysis of the change in cold tolerance (CT_min_), heat tolerance (CT_max_), egg-to-adult viability, and developmental time as a function of developmental temperature. We used sequential model reduction to find the minimal adequate model, such that model reductions were halted of predictors if they were part of a significant interaction. Effect sizes from the minimal adequate model are presented together with test statistics and significance level for the tested parameters. CT_min_ and CT_max_ were analyzed with linear models and *P* values were obtained with *F*-tests. Egg-to-adult viability and developmental time were both analyzed with generalized linear models with temperature as a quadratic term in which the significance of fixed effects was assessed with likelihood ratio tests. All model coefficients given are for males (except for egg-to-adult viability) and are thereby the deviation from the females. The second-degree term is given in parentheses. *P < 0.01; †*P* < 0.001.

#### NMR metabolomics: overall effects of developmental temperature and sex.

**Fig. 2.**
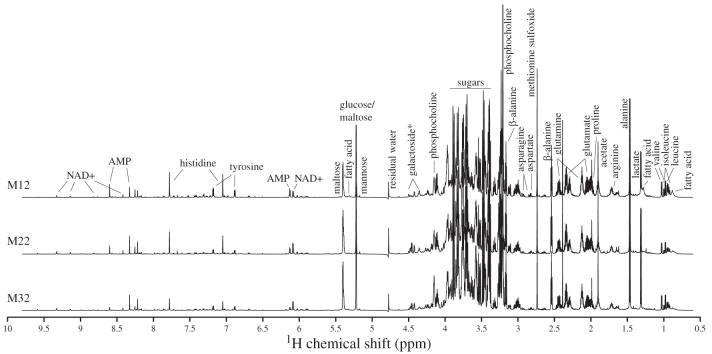
^1^H nuclear magnetic resonance (NMR) spectra of *Drosophila melanogaster* metabolites. NMR spectra of male flies after acclimation at 12, 22, and 32°C (M12, M22, and M32, respectively). The displayed spectra show the median intensities of all spectra in that temperature regime. Spectra were acquired with a Carr-Purcell-Meiboom-Gill (CPMG) delay of 194 ms at 25°C and sample pH was 7.4. *Full name of the galactoside is 1-*O*-(4-*O*-(2-aminoethyl phosphate)-β-d-galactopyranosyl)-glycerol.

**Fig. 3.**
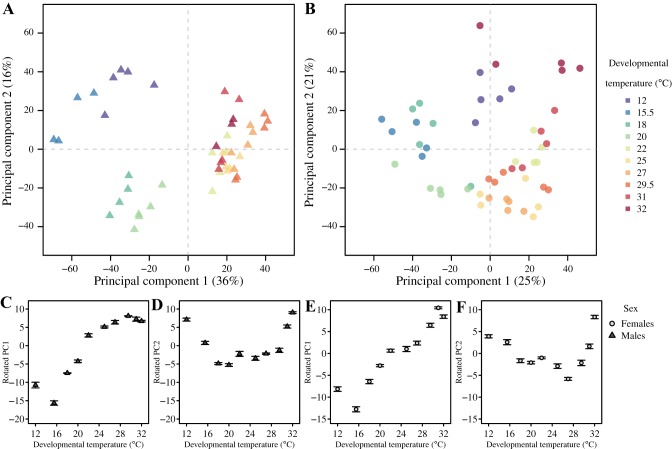
Principal component analysis (PCA) scores describing the overall metabolite variation across temperatures and sexes. *A*: male flies; *B*: female flies. Note that the axes in *A* and *B* do not necessarily represent the same metabolite changes. Below each PCA plot we have displayed rotated scores for males (*C* and *D*) and females (*E* and *F*). Rotations were performed such that the first rotated component correlated as much as possible with the developmental temperature (linear component) and the next component explained as much as possible of the remaining variation (u-shape component). These scores represent the norm of reaction for the change in concentration across developmental temperatures for groups of correlated metabolites. The correlation of the rotated scores with different metabolites is presented in Fig. 7.

Examples of metabolite NMR spectra of male flies acclimated at three different temperatures are presented in Fig. 2. To characterize the overall metabolite response to developmental temperatures in male and female flies, respectively, metabolite NMR spectra were analyzed by principal component analysis (PCA) (Fig. 3, *A* and *B*). Visual inspection of the PCA score plots reveals large variations in principal components 1 and 2 (PC1 and PC2) due to developmental temperatures for both sexes, but only segments of these are linear, and neither PC1 nor PC2 are linearly correlated with developmental temperature (Fig. 3, *A* and *B*). The overall grouping of samples can be described as circular. Thus samples from flies developed at similar temperatures appear close to each other (Fig. 3, *A* and *B*). But there is also a notable similarity between female flies reared at the maximum developmental temperature (32°C) and female flies reared at the minimum developmental temperature (12°C) (Fig. 3*B*), and this congruence was also apparent (though not to the same extent) in the male flies (Fig. 3*A*). To make some general conclusions on the overall metabolite change across developmental temperatures, we rotated the scores from the sex-specific PCAs such that the first rotated component showed as high a correlation as possible with developmental temperature (linear component) (Fig. 3, *C* and *E*). For both sexes, removal of the linear variation resulted in the next component having a distinct U-shape (U-shape component) (Fig. 3, *D* and *F*), indicating a dependence on the deviation from the intermediate temperature. The individual metabolites underlying these linear and U-shaped reaction norms of the overall metabolome are investigated below.

**Fig. 4.**
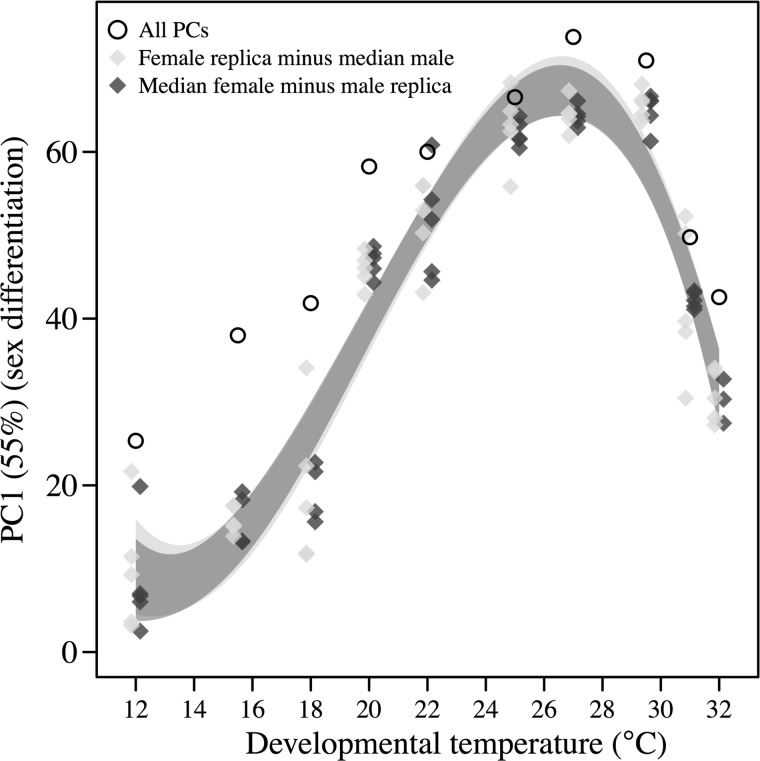
Sex differentiation in the metabolome across developmental temperatures**.** PC1 scores from a PCA based on the difference between individual female sample spectra and the median male spectrum at each temperature as well as the difference between median female spectrum and individual male sample spectra at each temperature. Higher scores in PC1 indicate a stronger differentiation between male and female samples, as the metabolite intensities were not centered. For comparison, the total score length across all averaged PCs (square roots of sum of squares) are also plotted for each developmental temperature. Minimal adequate models from the statistical analyses are plotted with the shaded area representing the 95% confidence interval.

We also performed a PCA on the spectral differences between male and female samples to investigate how the differentiation between sexes varies with developmental temperature. PC1 represents the majority of the sexual differentiation (Fig. 4) and showed a significant curved reaction norm across developmental temperatures (female replica minus median male: *F*_(3,42)_ = 181.84, *P* < 0.001; median female minus male replica: *F*_(3,45)_ = 140.92, *P* < 0.001). Inspection of the resulting 95% confidence intervals revealed that the lowest differentiation between sexes (low scores at PC1) occurs at low and high developmental temperatures (Fig. 4). Conversely, there is large differentiation at benign temperatures.

**Fig. 5.**
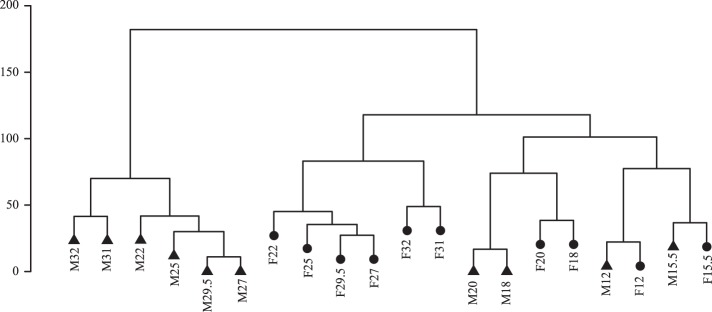
Similarities between combinations of developmental temperatures and sex. The grouping at the dendrogram illustrates circumstances where the effect of sex on the metabolome was stronger than the effect of developmental temperature (22–32°C) and vice versa (12–20°C). The dendrogram was derived by hierarchical cluster analysis of O2PLS-DA scores. The length of the vertical axis is a measure of the dissimilarities between clusters/groups of treatments. Males are displayed with triangles, whereas females are displayed with circles. In the tip labels F/M indicate female/male, and the following number indicates the developmental temperature.

O2PLS-DA in combination with HCA was used to illustrate the overall variation between flies at different temperatures and sexes in a dendrogram (Fig. 5). The dendrogram shows that the effect of developmental temperature on the metabolome is stronger than the effect of sex only at the lowest temperatures (12–15.5°C) (Fig. 5). At 17–20°C males and females form separate groups, but within the same cluster. Interestingly, flies reared at intermediate to high temperatures (22−32°C) form separate clusters for males and females, in which females cluster with flies reared at lower temperatures and males form a cluster on their own (Fig. 5).

#### NMR metabolomics: predicting phenotypes.

**Fig. 6.**
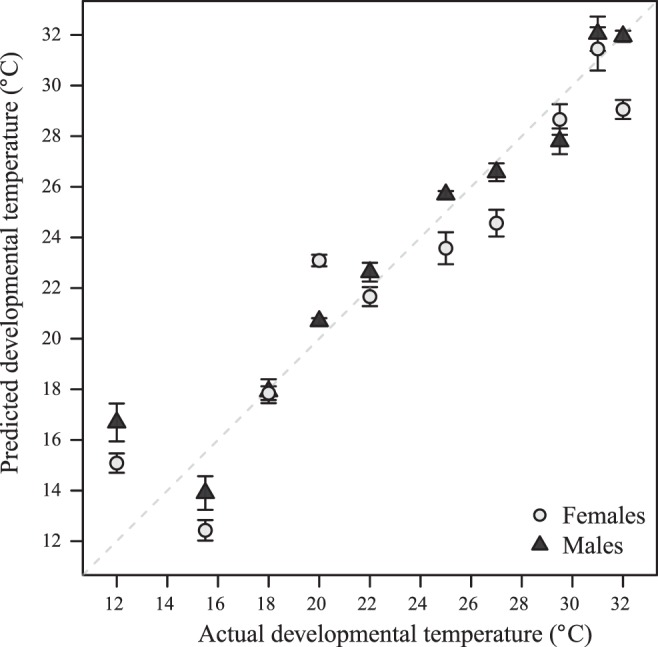
Prediction of developmental temperature from the metabolome**.** Orthogonal projections to latent structures (OPLS) model of temperature regime based on male and female flies. The predicted values are calculated using cross validation with all measurements for one condition left out from the model calculation when predicting the temperature regime for that condition. The predictabilities of the models (*Q*^2^) are 0.90 and 0.84 for males and females, respectively (Table 3). Positions were calculated as medians. Error bars represent 1 mean ± SE (*n* ≤ 5).

To assess the ability to predict the developmental temperature, extreme temperatures, developmental time, and egg-to-adult viability from metabolite data, we performed OPLS modeling. Egg-to-adult viability was evaluated with metabolite changes merged for males and females as no sex-specific egg-to-adult viability estimates were obtained. As shown in Table 3 all models were significant based on the total predictability *Q*^2^ (*Q*^2^ ≥ 0.5 is considered significant), providing statistical support for a set of metabolites with linear reaction norms across developmental temperatures, as well as a set of metabolites with inverted U-shaped reaction norms across developmental temperatures. Both for males and females the developmental temperature can be accurately predicted (*Q*^2^ = 0.90 and *Q*^2^ = 0.84, respectively) using a three-component linear model (linear prior) (Fig. 6). In comparison, the discrimination between extreme temperatures and those of the intermediate temperatures (U-shape prior) was not as accurate, although statistical significant. Finally, developmental time and egg-to-adult viability could also be predicted from the metabolome, but again not with the same accuracy as the developmental temperature.

**Table 3.** OPLS model statistics for parameter prediction from metabolite data

Predicted parameter	Metabolome	*A*†	*N*‡	*R*^2^§	*Q*^2^*
Linear prior	Male	1+2	46	0.58	**0.90**
	Female	1+2	50	0.46	**0.84**
U-shape prior	Male	1+1	46	0.48	**0.68**
	Female	1+1	50	0.33	**0.73**
Developmental time	Male	1+2	46	0.58	**0.71**
	Female	1+3	50	0.64	**0.60**
Egg-to-adult viability	Both	1+2	96	0.51	**0.61**

Capability of the *D. melanogaster* metabolome to predict the environmental parameter developmental temperature was tested using orthogonal projections to latent structures (OPLS) models. The test was done both with developmental temperature as a continuous parameter (linear prior), as well as a categorical parameter, that discriminates the two highest and lowest temperatures (12, 15.5, 3,1 and 32°C) from the intermediate temperatures (U-shape prior). We also assessed the predictability of developmental time and egg-to-adult viability. All parameters except egg-to-adult viability were predicted separately for each sex.

† *A* describes the number of model components where the first number accounts for the predictive component(s) correlating with the predicted variable and the second the orthogonal component(s).

‡ *N* describes the number of observations included in the model.

§ *R*^2^ describes how much of the total metabolite variation that is explained by the model.

* *Q*^2^ represents the predictability of the total model and is related to the statistical validity of the model. *Q*^2^ ≥ 0.5 is considered significant and is bold in the table. *Q*^2^ was calculated using cross-validation with all measurements for one condition left out at a time.

#### NMR metabolomics: individual metabolites.

**Fig. 7.**
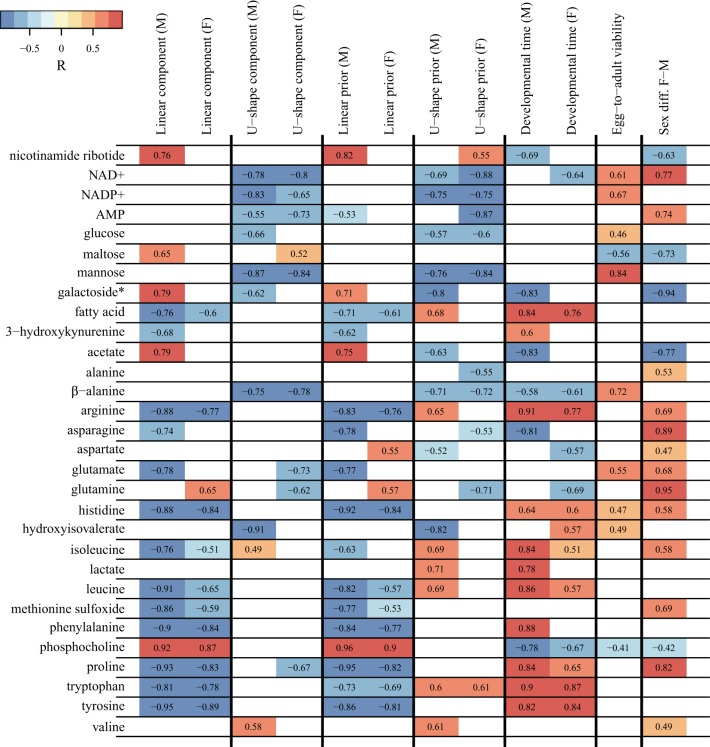
Metabolite changes correlated to temperature regime and survival characteristics. We identified reaction norms of individual metabolites across developmental temperatures using two hypotheses derived from rotated components of sex-specific PCAs of the metabolome as well as two prior hypotheses. We calculated the correlation coefficient (*R*) between individual metabolite intensities and the linear component, which describes the PCA component rotated to show the highest correlation with developmental temperature (see Fig. 3, *C* and *E*), and the U-shape component, which was obtained as the second component after removing the linear variation (see Fig. 3, *D* and *F*). Reaction norms following our earlier hypotheses were tested by correlating individual metabolite intensities with developmental temperature (linear prior) and with a categorical variable discriminating the two highest and lowest temperatures (12, 15.5, 31, and 32°C) from the intermediate temperatures (U-shape prior). The tests of these prior hypotheses, as well as the correlations to phenotypic traits developmental time and egg-to-adult viability, were calculated as the correlation coefficient (*R*) with OPLS model scores for the respective parameters. Egg-to-adult viability was evaluated for males and females together as there were no sex-specific egg-to-adult viability estimates. Sex diff. F-M is the first component in a PCA of the spectral differences between male and female samples (see Fig. 4), whereby significant correlations with this parameter indicate that the given metabolite contributes to the sexual dimorphism observed in the metabolome. Significant correlations with the linear component and/or the linear prior shows evidence of an approximate linear norm of reaction of the given metabolite across developmental temperatures, with a negative correlation coefficient representing a decrease in concentration with increasing temperatures and vice versa. Conversely, a significant correlation with the U-shape component and/or the U-shape prior illustrates that the change in concentration from intermediate temperature to high and low temperatures is in the same direction. Here a negative correlation coefficient represents a decrease in concentration at extreme temperatures (bell-shaped reaction norm), whereas a positive correlation coefficient represents an increase in concentration at extreme temperatures (U-shaped reaction norm). Significant spectral correlations were identified by applying sequential Bonferroni correction (*P* < 0.05) for an assumed total number of 100 metabolites. Only significant correlations are presented. Note that although this procedure minimizes the false positives, it does nothing to limit the false negatives. *Full name of the galactoside is 1-*O*-(4-*O*-(2-aminoethyl phosphate)-β-d-galactopyranosyl)-glycerol.

The individual metabolite variation forming the basis for the linear and U-shaped reaction norms are shown in Fig. 7. Overall, metabolites that correlate with the linear prior are largely identical with the metabolites correlating with the linear component, whereas metabolites that correlate with U-shape prior also correlate with U-shape component. Metabolites correlating with the linear terms were dominated by a decrease in amino acids with increasing developmental temperature. Ten metabolites (arginine, histidine, isoleucine, leucine, methionine sulfoxide, phenylalanine, proline, tryptophan, tyrosine, and fatty acid) decreased linearly in both males and females, while only phosphocholine increased. Additionally, male flies showed an increase in nicotinamide ribotide, maltose, acetate, and the galactoside 1-*O*-(4-*O*-(2-aminoethyl phosphate)-β-d-galactopyranosyl)-glycerol and decreases in 3-hydroxykyneurenine and glutamate. In females, the only additional linear effect was an increase in glutamine. The U-shaped terms were dominated by changes in energy-carrying molecules that were decreased at extreme temperatures (showing the reverse behavior of the scores displayed in Fig. 3, *D* and *F*). Five metabolites showed this inverted U-shape response in both males and females: NAD+, NADP+, AMP, mannose, and β-alanine. Males showed additional changes along the same axis in glucose, the galactoside, and hydroxyisovalerate, and in the opposite direction in isoleucine and valine. Females showed additional changes in maltose, glutamate, glutamine, and proline. No metabolite showed significant changes in opposite directions in males and females.

The individual metabolite changes that correlated with developmental time and viability are also shown in Fig. 7. The metabolites that correlated with the linear terms also correlate with developmental time, except for a reversal of the sign of the correlation coefficient (Fig. 7). Egg-to-adult viability shared a high number of metabolites with the U-shaped terms, but with a reversal of the sign of the correlation coefficient (Fig. 7).

## DISCUSSION

How organisms perceive and adapt to changes in temperature is central for our understanding of species ecology, species distributions, and ecological networks. Numerous studies have used metabolomics to investigate associations between temperature and metabolite composition in ectotherms (10, 19, 32, 37, 39, 45). Here we investigated functional phenotypes and the metabolome in female and male flies from the developmental thermal range that *D. melanogaster* can tolerate, enabling estimation of reaction norms of groups of metabolites. The resulting relationship between metabolites and the functional phenotypes allows for an improved understanding of how organisms respond and adapt to cope with thermal stress via ecophysiological interactions.

### 

#### Functional phenotypes.

We propose that the functional phenotypes support the interpretation of the metabolomic results, whereby their detail and generality across species deserves some attention. The life-history data (egg-to-adult viability and developmental time) are in line with previous studies (14, 42, 57) and provide detailed information on consequences of developmental temperatures on fitness components. Our finding that upper thermal limits are less plastic than lower thermal limits is also in line with previous findings (22, 61). Thus studies investigating reaction norms of thermal limits in 13 species of *Drosophila* (58), as well as studies on fish (31) and lizards (9), lead to the same conclusions. We also note that the linear increase of CT_min_ with increasing temperatures is a characteristic shared across *Drosophila* species, whereas the slope of the linear increase of CT_max_ is highly species specific (58). This linearity suggests that the physiological mechanisms involved in the acclimation are continuously adjusted, instead of an “edge” effect, in which a temperature threshold facilitates a given physiological mechanism, promoting a large change in thermal tolerance within a small temperature range. It is well known that males develop slower than females across the entire thermal range (46). Less well established are the characteristics of the sexual dimorphism that may exist in the reaction norms of upper and lower thermal limits. We here show that the sexual dimorphism in CT_min_ is very small, if present at all. Conversely, females had consistently higher CT_max_ than males, and with some indication that this difference was largest at the lowest temperatures.

#### Metabolome and relationship with functional phenotypes.

As for the functional phenotypes, we also found large effects of the developmental temperature on the metabolome. The majority of these effects were directional across developmental temperatures, in accordance with these metabolites being a component of the physiological thermal response of the organism (*hypothesis 2*). This group of metabolites mainly consisted of amino acids that increased with decreasing developmental temperatures, a pattern that was consistent across sexes (Figs. 3 and 7). The increase in free amino acids at lower temperatures is in accordance with earlier studies on *D. melanogaster* and *Cydia pomonella* (10, 32, 52), including proline, which has been shown to be important for cold acclimation in several insect species (33, 34). We did not observe any consistent change in alanine, which has been related to heat and cold shock responses (39, 43) but also seems to be less important for *Drosophila* species than in other insects (47). Glutamate and glutamine have been related to the heat shock response in *D. melanogaster* (39), but we did not find a consistent pattern for these metabolites, and it is therefore likely that their regulation is unrelated to long-term heat acclimation. Phosphocholine stood out as the only metabolite increasing at higher developmental temperatures in both sexes, whereas the galactoside, which exclusively occurs in males (45), also increased at higher temperatures (Fig. 7). Phosphocholine has not been connected to thermal acclimation before, and given the strong correlation we show here, this makes it an interesting new candidate metabolite involved in thermal acclimation. Phosphocholine is an intermediate of phosphatidylcholine, a class of phospholipids and a well-known component of cell membranes. The increase of phosphocholine at higher temperatures is likely a result of restructuring of biological membranes as a part of thermal acclimation, but studies directed toward this metabolite are needed for further hypothesis development. CT_min_ and CT_max_ both correlated linearly with developmental temperature, making a correlation between these tolerance traits and the metabolome redundant in terms of identifying metabolites directly related to thermal resistance. The metabolite data presented characterize the constitutive metabolome and does not reflect the changes that occur up until the point of critical temperature determination during the thermal tolerance assays. Nevertheless, it can be concluded that these metabolites are part of a large set of physiological mechanisms enabling insects to adapt plastically to diurnally and seasonally changing thermal environments. It also shows that the inherent structures of the metabolite changes and their temperature dependence share strong similarities with the temperature dependence of the functional phenotypes.

An increase in sugars with decreasing developmental temperatures has been reported in several studies, and there is a consensus that these are important for long-term (10, 32) as well as short-term cold acclimation (43, 64). Central sugars from the above studies, such as fructose and trehalose, were not identified in the current study, but we found both mannose and, to a lesser extent, glucose to decrease at both extreme low and high developmental temperatures (Fig. 7). This pattern is unexpected, but confirms earlier suggestions of sugars to be related to a thermal stress response (10), and thus not only connected to cold acclimation but to thermal stress in general. Mannose and glucose were part of a group of metabolites showing an inverse U-shaped norm of reaction across temperatures (Figs. 3 and 7). These metabolites were also detected when we identified metabolites related to the nonlinear norm of reaction of egg-to-adult viability (Figs. 1 and 7). Consistently across sexes, this group of metabolites, which is primarily related to energy metabolism (NAD+, NADP+, and AMP), was depleted at the lowest and highest temperatures. We interpret this group, together with the depleted sugars, as a set of “cost” metabolites that characterize a departure from cellular homeostasis, and thus challenges the acclimation of the organism at the edges of the investigated environmental gradient (*hypothesis 3*). These molecules are potential candidates for molecular estimators of cellular thermal stress or stress in general and constitute an example of a commonality between high and low temperature stress. Such similarities indicate that apart from the commonly observed low and high temperature-specific responses (4, 36), shared cellular challenges during cold and heat stress also exist. Indeed, results from selection experiments show how flies selected for increased heat resistance are both more heat and cold resistant (5), whereas a short-term heat shock before cold shock can increase cold resistance (6).

#### Sex effects on metabolome.

In general, the effect of sex on the metabolome was stronger than the effect of developmental temperature, except at low rearing temperatures (Fig. 5). This was supported by the PCA on metabolomic sex differences, which provides evidence for a peak in sex differentiation at intermediate temperatures, intermediate differentiation at high temperatures, and low differentiation at low temperatures (Fig. 4, see Fig. 7 for individual metabolites). The decrease in differentiation between males and females at extreme temperatures is not consistent with observations on thermal limits and thus likely driven by the common metabolomic response to stress at extreme temperatures. Strong sex effects on the metabolome have previously been identified (21). We provide evidence that supports this conclusion but also show that the metabolomic differences between males and females are highly dependent on developmental temperature and thus environment specific (Fig. 4). Male- and female-specific acclimation responses have previously been observed in ectotherms (21, 63). Such dimorphism can lead to different selection intensities in males and females exposed to stressful temperatures and ultimately lead to sex-specific life-history evolution as the degree of plasticity can impact on the strength of selection (51, 72). Our data pinpoint traits and physiological mechanisms, which in variable thermal environments, are likely to be under different selection pressures in males and females.

### Perspectives and Significance

The presented NMR data provide novel insights into the underlying physiology of the large effects of developmental temperature observed in the functional phenotypes. We found developmental temperature to be a strong predictor of the metabolome; however, this association was sex dependent, especially at higher developmental temperatures. Evidence that the metabolome is a sensitive indicator of physiological state and biological age in *D. melanogaster* has previously been reported (21, 53). We show here that the metabolomic fingerprint also accurately predicts developmental temperatures by a mixture of “beneficial” metabolites related to the functional phenotypes cold and heat tolerance and “cost” metabolites with signatures associated with the fitness phenotype egg-to-adult viability through energy-carrying molecules.

## GRANTS

This research was funded by the Graduate School of Science and Technology at Aarhus University to M. F. Schou, by the Danish Natural Science Research Council with a frame grant to V. Loeschcke, by a Sapere aude stipend to T. N. Kristensen (DFF-4002-00036), and by the Department of Biomedical Sciences at the University of Copenhagen to A. Malmendal.

## DISCLOSURES

No conflicts of interest, financial or otherwise, are declared by the author(s).

## AUTHOR CONTRIBUTIONS

M.F.S. and T.N.K. conceived and designed research; M.F.S., T.N.K., A.P., G.K., and A.M. performed experiments; M.F.S., T.N.K., and A.M. analyzed data; M.F.S., T.N.K., and A.M. interpreted results of experiments; M.F.S. and A.M. prepared figures; M.F.S. and T.N.K. drafted manuscript; M.F.S., T.N.K., V.L., and A.M. edited and revised manuscript; M.F.S., T.N.K., G. K., V.L., and A.M. approved final version of manuscript.
